# Podbat: A Novel Genomic Tool Reveals Swr1-Independent H2A.Z Incorporation at Gene Coding Sequences through Epigenetic Meta-Analysis

**DOI:** 10.1371/journal.pcbi.1002163

**Published:** 2011-08-25

**Authors:** Laia Sadeghi, Carolina Bonilla, Annelie Strålfors, Karl Ekwall, J. Peter Svensson

**Affiliations:** Department of Biosciences and Nutrition, Karolinska Institutet, Huddinge, Sweden; University of California San Diego, United States of America

## Abstract

Epigenetic regulation consists of a multitude of different modifications that determine active and inactive states of chromatin. Conditions such as cell differentiation or exposure to environmental stress require concerted changes in gene expression. To interpret epigenomics data, a spectrum of different interconnected datasets is needed, ranging from the genome sequence and positions of histones, together with their modifications and variants, to the transcriptional output of genomic regions. Here we present a tool, Podbat (Positioning database and analysis tool), that incorporates data from various sources and allows detailed dissection of the entire range of chromatin modifications simultaneously. Podbat can be used to analyze, visualize, store and share epigenomics data. Among other functions, Podbat allows data-driven determination of genome regions of differential protein occupancy or RNA expression using Hidden Markov Models. Comparisons between datasets are facilitated to enable the study of the comprehensive chromatin modification system simultaneously, irrespective of data-generating technique. Any organism with a sequenced genome can be accommodated. We exemplify the power of Podbat by reanalyzing all to-date published genome-wide data for the histone variant H2A.Z in fission yeast together with other histone marks and also phenotypic response data from several sources. This meta-analysis led to the unexpected finding of H2A.Z incorporation in the coding regions of genes encoding proteins involved in the regulation of meiosis and genotoxic stress responses. This incorporation was partly independent of the H2A.Z-incorporating remodeller Swr1. We verified an Swr1-independent role for H2A.Z following genotoxic stress *in vivo*. Podbat is open source software freely downloadable from www.podbat.org, distributed under the GNU LGPL license. User manuals, test data and instructions are available at the website, as well as a repository for third party–developed plug-in modules. Podbat requires Java version 1.6 or higher.

This is a *PLoS Computational Biology* Software Article.

## Introduction

Epigenetic regulation consists of a multitude of different inter-connected modifications that determine the active and inactive states of chromatin. Chromatin is modified in a variety of ways, such as post-translational histone modifications and reorganization of histone variants, but also DNA methylations and control of the DNA helical torsion [1], [2]. Pivotal cellular changes such as those that occur during cellular differentiation, cell cycle arrest or programmed cell death induced by genotoxic stress, require prompt and accurate changes of gene expression patterns. The co-dependency of chromatin modifications makes it desirable to analyze the entire range of modifications simultaneously and, in addition, relate it to transcriptional levels [3]. In fission yeast, *Schizosaccharomyces pombe*, chromatin regulation has been widely studied using epigenomics techniques. However, as studies have used different platforms, it is challenging to combine datasets without loosing the details of the original data. The large data volume from genomic experiments makes the analysis cumbersome even for model organisms with relatively small genomes. Several tools are available to visualize the data [4], [5], [6] and commercial software (Partek Inc., St Louis, MO; DNAnexus Inc., Palo Alto, CA; GeneSpring GX, Agilent Technologies Inc., Santa Clara, CA; Avadis NGS, Strand Scientific Intelligence, San Fransisco, CA) is useful for analysis, and usually cannot easily be appended for researcher-generated questions. Especially, few algorithms can be used to identify regions where proteins are bound to DNA in a multi-sample dataset. In combination, this generally results in the need of informatics support and *ad hoc* script implementation to explore the experimental data.

We set out to develop an integrated computational tool, Podbat (Positioning database and analysis tool) for use on epigenomics datasets. Podbat is freely available and open source, focuses on scientific clarity and user-friendliness, and implements robust and cutting-edge algorithms. We exemplify the use of Podbat by reanalyzing all to-date published genome-wide datasets for genome occupancy data of the histone variant H2A.Z in *S. pombe* in WT and *swr1*Δ cells [7], [8], [9], [10] together with transcriptional data after different perturbations [9], [11], [12], [13], [14], [15], [16], [17], [18], [19] (Table 1). To exemplify the possibility of cross-species comparisons, datasets from the related budding yeast, *Saccharomyces cerevisiae*, is included in the analysis [20], [21]. H2A.Z is encoded by the *pht1^+^* gene in *S. pombe* [22], and is a replacement variant of the core histone H2A. It is known that H2A.Z is incorporated in the nucleosomes close to the promoters by chromatin remodeler Swr1 [23], [24], marking genes poised for activation [25]. However, recently, H2A.Z has been identified as playing a role in transcription elongation [26], [27] and also in Swr1-independent functions following genotoxic insult [28]. The Podbat analysis presented here led to previously unexpected findings regarding H2A.Z-mediated gene regulation.

**Table 1:** Genome-wide data sources used for this study.

Data	Type	Platform	Study
*Schizosaccharomyces pombe*			
H2A.Z, *pht1Δ*, *swr1Δ*	ChIP-chip, RNA	single color microarray	Buchanan et al, 2009 [7]
H2A.Z, *pht1Δ*, *swr1Δ*	ChIP-chip, RNA	dual color microarray	Zofall et al, 2009 [10]
*pht1Δ*, *pht1ΔN*, *swr1Δ*	RNA	dual color microarray	Kim et al, 2009 [9]
H3K4me, H3K9me	ChIP-chip	single color microarray	This study
Mitosis	RNA	dual color microarray	Rustici et al, 2004 [19]
Mitosis	RNA	dual color microarray	Peng et al, 2005 [16]
Mitosis	RNA	dual color microarray	Oliva et al, 2005 [15]
Meiosis	RNA	dual color microarray	Mata et al, 2002 [12]
Starvation	RNA	dual color microarray	Murai et al, 2009 [18]
Ste11p	RNA	dual color microarray	Mata & Bähler, 2006 [14]
Environmental stress	RNA	dual color microarray	Chen et al, 2003 [11]
Environmental stress	RNA	single color microarray (HybMap)	Dutrow et al, 2008 [17]
Cisplatin response	RNA	dual color microarray	Gatti et al, 2004 [13]
*Saccharomyces cerevisiae*			
H2A.Z, H2A.Z-Lys14	ChIP-chip	dual color microarray	Millar et al, 2006 [21]
Environmental stress	RNA	dual color microarray	Gasch et al, 2000 [20]

## Design and Implementation

### Podbat Description

**Figure 1:**
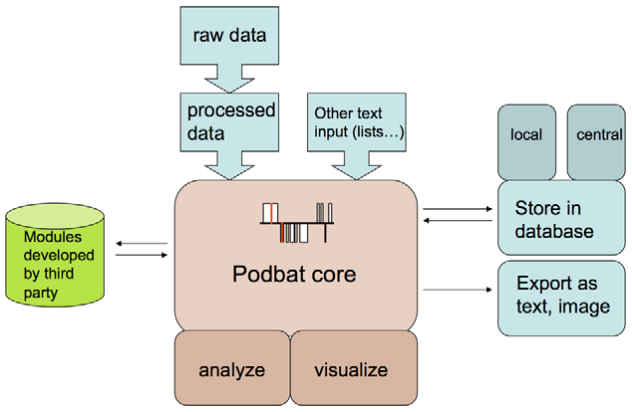
Schematic view of the architecture of Podbat. The controller of the core component is the hub of the application. The core handles input and output from data files and independent modules. Text files with genomic data are imported into Podbat.

In order to interpret epigenomics data, Podbat follows a simple flowchart (Figure 1) and implements a flexible genome browser in its core. The main view of the user interface (Figure S1) consists of three core panels: 1) the ‘genome panel’ that displays the chromosomes and the selected regions together with the associated data in a visual manner; 2) the ‘selected element panel’, displaying a spreadsheet with the elements – genes, siRNA, promoter regions or any other element of interest – together with associated data in numerical or text format; 3) the ‘control panel’ where the user can preprocess data, define and apply filters and create sets of selected genetic elements.

**Figure 2:**
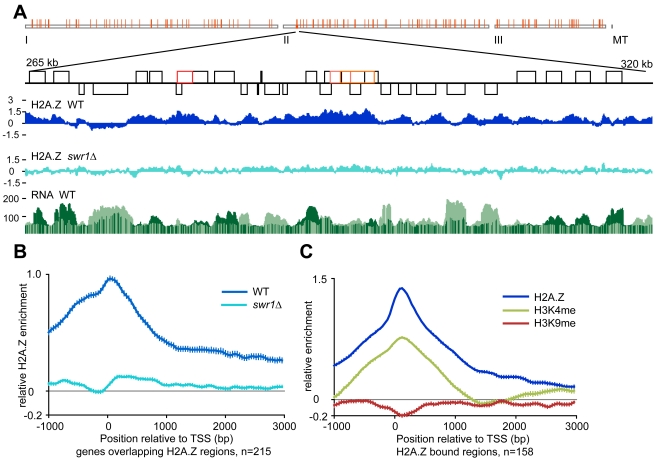
H2A.Z is mainly found in promoter regions and coincides with active marks of transcription. A) Regions (red squares) with high levels of H2A.Z are identified using Hidden Markov Models. Many of the promoter regions show some H2A.Z occupancy. Top panel displays the genomic overview, bottom panel shows a zoomed in fraction of the genome. ORFs are displayed as black boxes. B) The genome average shows that most H2A.Z is found at the promoter region of genes, and this is dependent on Swr1. The data is aligned at the Translation Start Site (TSS). C) The active mark H3K4me coincides with H2A.Z, but not the repressive mark H3K9me. Error bars represent the 95% confidence interval.

Podbat was optimized using the yeast genomes of *S. cerevisiae* and *S. pombe*. Both genomes are relatively compact, 12 Mb and 13 Mb respectively. However, any organism with a sequenced genome can be uploaded. Genomes can easily be imported and updated as Podbat connects directly to Ensembl, facilitating the use of the latest available annotations. Once the genome is set, experimental data is loaded and superimposed. Data of different sources may be visualized and analyzed simultaneously. Files can be uploaded in standard formats used for sequencing and microarray experiments. Once the data is loaded, regions can be identified based on the input data (Figure 2A). Two algorithms are available for this purpose: i) Hidden Markov Models (HMM) and ii) sliding windows. The parameters can be estimated *ad hoc* or using the Baum-Welch algorithm [29]. Automated estimation of the parameters leads to robust classification results [30]. As chromatin immunoprecipitation (ChIP) experiments generate DNA fragments of considerable length (typically >100 bp), regions defined in different samples will be variable by nature. By consecutive determination of regions in all samples, regions can be merged to detect biologically relevant fragments. When analyzing RNA data, this allows the detection of all transcribed regions, even if not previously described or in genomes where annotations are not available. The regions can be quantified – reflecting the expression of genes or binding to genetic elements, filtered and compared between experiments. The output is provided in a spreadsheet format for further analysis.

Additionally, Podbat allows data alignments of gene sets by the start or end position (Figure 2B–C). Using this functionality, one can visualize e.g. a protein binding within region surrounding a fix position, such as the start codon. To analyze different sets of genes, tests of enrichment can be performed between gene sets. This is a powerful method to find correlations between and within datasets. Gene sets can be defined manually from previous experiments or from public databases such as GO (Gene Ontology) or KEGG (Kyoto Encyclopedia of Genes and Genomes).

### Data Storing and Sharing

In addition to saving sessions, the user has the option to store and share datasets by exporting the data into a MySQL database. The database connects seamlessly with the Podbat user interface. For unpublished data, users can create a local database, only accessible for data sharing within a smaller community. To publicly share data, a central database can be accessed. By public or private central data storage, the software facilitates data meta-analysis and incorporation of data from previous experiments. Several datasets for histone modifications and RNA transcription in various mutants of *S. pombe* are currently centrally uploaded and available for comparison.

### Podbat Modules

Podbat functionality can be added by third party developed plug-in modules. These modules may extend the methods of the application core and tailor analysis. Development guidelines and Podbat application programming interface (API) are available at http://www.podbat.org. Validated and tested plug-in modules may be accommodated on this webpage.

## Results

### Swr1 Rearranges Nucleosomes and Incorporates H2A.Z as an Active Mark

*S. pombe* and *S. cerevisiae* H2A.Z occupancy data from different sources and four experimental platforms was imported into Podbat (Table 1) [7], [10], [21]. We show here the results from one of the *S. pombe* datasets, but the other data is consistent with these results (Supplemental material). As expected, most genes had an Swr1-dependent H2A.Z peak near the first transcribed nucleosome (Figure 2A,B). The average value representing the enrichment of the H2A.Z binding for these regions was 0.76±0.25 (mean±s.d.), which became reduced to 0.04±0.17 in the *swr1Δ* strain. The genome wide enrichment of H2A.Z in WT was 0.06±0.45 at ORFs. We identified 157 genomic regions of H2A.Z occupancy of length>100 bp and at least 2.5 fold increase over background through HMM (Table S1). HMM parameters were estimated by the Baum-Welch algorithm [29]. The H2A.Z mark coincides with methylation of histone H3 lysine 4 (H3K4me), a mark of active chromatin, but does not correlate with the repressive mark of methylated lysine 9 (H3K9me) (Figure 2C). The regions of enriched H2A.Z occupancy overlap with 214 genes (Table S2). The Gene Ontology annotations of these genes showed enrichment for two broadly defined categories: cell cycle maintenance (both mitotic and meiotic) and DNA damage response. Also genes involved in transport, mitochondrial function and structure as well as metabolism were overrepresented among the H2A.Z bound regions (Table 2 and Table S3).

**Table 2:** Enriched Gene Ontology Annotations.

GO Term	Description	Fold Change	P-Value
GO:0042802	identical protein binding	4.7	0.0002
GO:0004540	ribonuclease activity	3.7	0.0005
GO:0000077	DNA damage checkpoint	4.1	0.0005
GO:0042770	signal transduction in response to DNA damage	3.9	0.0007
GO:0000075	cell cycle checkpoint	2.7	0.0010
GO:0008033	tRNA processing	2.9	0.0011
GO:0000001	mitochondrion inheritance	8.0	0.0014
GO:0006479	protein methylation	3.2	0.0015
GO:0008168	methyltransferase activity	2.7	0.0015
GO:0006839	mitochondrial transport	2.6	0.0021
GO:0007131	reciprocal meiotic recombination	3.2	0.0026
GO:0007059	chromosome segregation	2.1	0.0030
GO:0000723	telomere maintenance	2.9	0.0039
GO:0007126	meiosis	2.0	0.0040
GO:0009116	nucleoside metabolic process	3.2	0.0044
GO:0006310	DNA recombination	2.4	0.0047
GO:0031570	DNA integrity checkpoint	2.8	0.0047
GO:0007127	meiosis I	2.5	0.0050

P-value<0.005, more than 5 selected genes, enrichment > = 2.0.

### During the Cell Cycle, the H2A.Z Distribution Is Modified by Swr1

**Figure 3:**
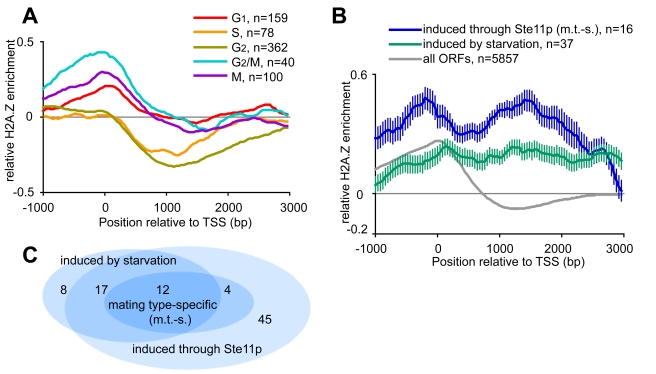
H2A.Z is differentially incorporated in gene coding regions of specific periods of the mitotic and meiotic cell cycle. A) Genes that are up-regulated in S and (early) G_2_ show H2A.Z depletion within the coding sequence (error bars not shown for clarity). B) Genes induced late after nitrogen starvation show a distribution of H2A.Z in gene coding regions. This effect is even more pronounced in the Ste11p-induced mating type specific genes. Error bars represent the 95% confidence interval. C) Venn diagram showing the overlap between genes induced after N_2_ starvation and genes under Ste11p control, including the subset which is mating type-specific (m.t.-s.).

To further examine the genome-wide H2A.Z occupancy, we consulted all published genome-wide data on cell cycle and stress response in *S. pombe* (Table 1). The data from previously identified gene sets were aligned at the Translation Start Sites (TSS). We noted that most gene sets deviated minimally from the average gene profile, i.e. H2A.Z occupancy was found in the promoter regions in an Swr1-dependent fashion [15], [16], [19]. Interestingly, cell cycle specific gene sets revealed that the genes up-regulated in S and early G_2_ phase of the cell cycle had an H2A.Z depletion in promoter regions and within the coding sequence (Figure 3A). This effect was not related to differential expression of the genes (data not shown). As for meiotic genes, one specific gene set deviated, consisting of genes induced late after nitrogen starvation, before diploid cells enter into meiosis [12], [18]. These genes had a pronounced Swr1-dependent H2A.Z occupancy in both promoters and gene coding sequences. This gene set is highly related to genes under transcriptional control by Ste11p (Figure 3C) [14], a transcription factor critical for regulation of mating-type specific genes during meiosis [31]. The subset of mating-type specific Ste11p-controlled genes showed an even more pronounced H2A.Z binding (Figure 3B).

### Swr1-Independent H2A.Z Incorporation Relevant to Genotoxic Stress Response

**Figure 4:**
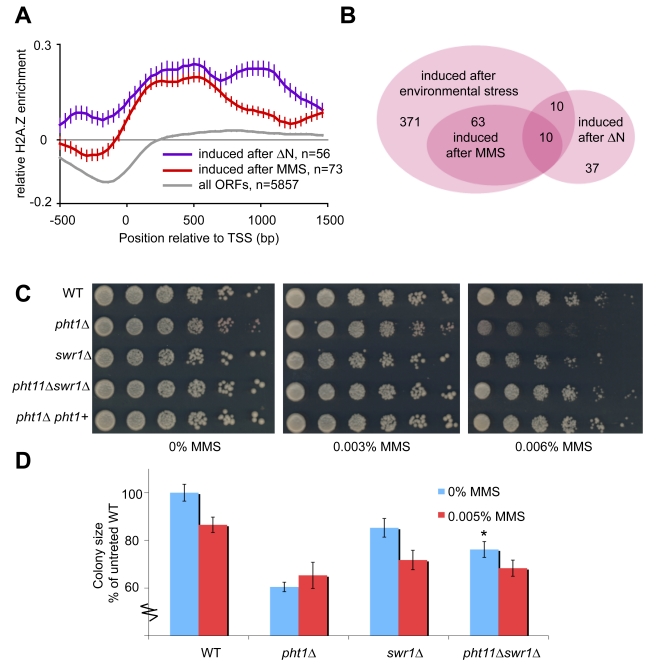
Genes induced by genotoxic stress have an Swr1-independent incorporation of H2A.Z. A) Genes induced by MMS have an Swr1-independent incorporation of H2A.Z in their gene coding regions. This is also true for genes that are up-regulated by deletion of the acetylatable N-terminal of H2A.Z (ΔN). Error bars represent the 95% confidence interval. B) Venn diagram showing the overlap between the different gene sets. C) Cells lacking H2A.Z (*pht1*Δ) have a slight sensitivity to high levels of MMS-induced genotoxic stress. Mutant and WT cells were diluted 1∶10, plated on YES with different doses of MMS (0%, 0.003%, 0.006%). D) Colony size determination reveals an SWR1-independent effect of deleting H2A.Z (*pht1*Δ) after treatment with MMS. Asterisk (*) marks a significant genetic interaction (p<0.001). At least 20 colonies were measured each in 5 independent experiments.

The coding region of stress-responsive genes [11], [13], [17] were associated with a slight but significant (p<10^−28^) H2A.Z occupancy in cells lacking Swr1 (data not shown). Also, analysis of H2A.Z deposition in *S. cerevisiae* revealed significant overlap of gene coding sequences bound by the H2A.Z homolog [21] and genes differentially transcribed after environmental stress [20] (Figure S4). Dissection of the stress response in *S. pombe* revealed that an Swr1-independent H2A.Z binding at gene coding regions was obvious after genotoxic stress (Figure 4A). Genes induced after exposure to MMS and cisplatin showed similar patterns. Recently, it was shown in *S. cerevisiae* that the roles of the Swr1-containing complex and H2A.Z become decoupled after treatment with MMS [28]. As cells are damaged, H2A.Z becomes deacetylated. We investigated the H2A.Z occupancy profile of genes that are affected by changes in acetylation of H2A.Z [9]. A large proportion of these genes are MMS-induced (Figure 4B). We found that genes that are up-regulated upon deletion of the acetylation residues (H2A.Z ΔN) follow the same pattern and have a strong Swr1-independent H2A.Z occupancy in gene coding regions (Figure 4A).

To determine whether the Swr1-independent H2A.Z functions are relevant for the cellular phenotype, we tested the sensitivity of mutant *S. pombe* cells to different conditions. Cells devoid of H2A.Z, Swr1 or both were exposed to MMS (Figure 4C). At a quick glance, *pht1Δ* (lacking H2A.Z) appears to be sensitive but even at high doses, many small colonies can be observed. Reintroduction of the *pht1^+^* gene rescues the phenotype. The *swr1Δ* and the *pht1Δswr1Δ* cells show the same sensitivity as WT. More importantly, determination of colony size under basal conditions and after MMS exposure (Figure 4D) reveals a suppressive genetic interaction between *pht1^+^* and *swr1^+^* (with a score +2.0, p<0.001, calculated as in [32]). However, as in the related yeast *S. cerevisiae*, this interaction is reduced in the MMS treated condition (score +1.4, p>0.05) [28], as expected from our computational analysis, confirming an Swr1-independent effect of H2A.Z after genotoxic damage.

In summary, the results presented here confirm an Swr1-independent pattern of H2A.Z distribution, relevant for the response to DNA damage.

## Availability and Future Directions

Podbat is open source software implemented as a desktop application in Java and can be freely and anonymously downloaded from www.podbat.org or as supplemental material accompanying this paper (Software S1). Podbat is distributed under the GNU LGPL license. User manuals, test data and instructions are available at the website, as well as a repository for third party developed plug-in modules. Podbat is tested and runs on any platform that supports Java version 1.6 and higher. Podbat is an ongoing project and new modules are continuously being developed. Data used to generate results presented in this article are available as supplementary information.

## Supporting Information

Figure S1 Screenshot of Podbat with 5 datasets loaded. Four genes on chromosome II are highlighted in red. A quantification of the signal has been determined of the protein binding/RNA expression/histone modification.
(TIF)

Figure S2 H2A.Z bound genes of different gene sets are aligned at the Translation Start Site (TSS) from the dataset GSM432595 from Zofall et al. [10]. H2A.Z bound genes were identified as overlapping with HMM determined regions of increased H2A.Z signal (parameters used as in the other datasets with the exception of Region length threshold = 20 instead of 100, since this array has probes more sparsely spaced). 94 regions were identified, overlapping with 438 genes. **A**) Genes differentially expressed during different stages of the cell cycle [16]. B) Gene induced by late after nitrogen starvation [12] and mating-type specifically (m.t.-s.) regulated genes induced through Ste11p [14].
(TIF)

Figure S3 H2A.Z bound genes of different gene sets are aligned at the Translation Start Site (TSS) from the dataset GSM432576 of the partial from Zofall et al. [10]. Only approximately 2 Mb of the genome (15%), consiting of chromosome II and three telomeric sequences (1L, 2L, 2R) are covered in this array. 994/5857 ORFs are within this region. H2A.Z bound genes were identified as overlapping with HMM determined regions of increased H2A.Z signal (parameters used as in the other datasets). 27 regions were identified, overlapping with 113 genes. **A**) Genes differentially expressed during different stages of the cell cycle [16]. B) Gene induced by late after nitrogen starvation [12] and mating-type specifically (m.t.-s.) regulated genes induced through Ste11p [14]. **NB** the small number of investigated genes in these gene set.
(TIF)

Figure S4 Venn diagrams showing the overlap between genes where the coding regions are bound by H2A.Z homolog in *S. cerevisiae* (levels deviating 2-fold from genome average) and genes differentially regulated after environmental stress. P-values for overlaps are calculated by the hypergeometric distribution.
(TIF)

Software S1 Podbat software.
(TAR)

Table S1 H2A.Z bound regions.
(XLS)

Table S2 H2A.Z bound genes.
(XLS)

Table S3 Complete list of GO terms enriched in the H2A.Z bound genes from [7].
(XLS)

Table S4 GO terms enriched in the GSM432595 data [10] of H2A.Z bound genes.
(XLS)

Table S5 GO terms enriched in the GSM432576 data [10] (querying partial genome) of H2A.Z bound genes.
(XLS)
