# Supplementary figures and images for: Podbat: A Novel Genomic Tool Reveals Swr1-Independent H2A.Z Incorporation at Gene Coding Sequences through Epigenetic Meta-Analysis
Source: PLoS Comput Biol. 2011 Aug 25;7(8):e1002163. doi: 10.1371/journal.pcbi.1002163 (PMC3161910; doi:10.1371/journal.pcbi.1002163)

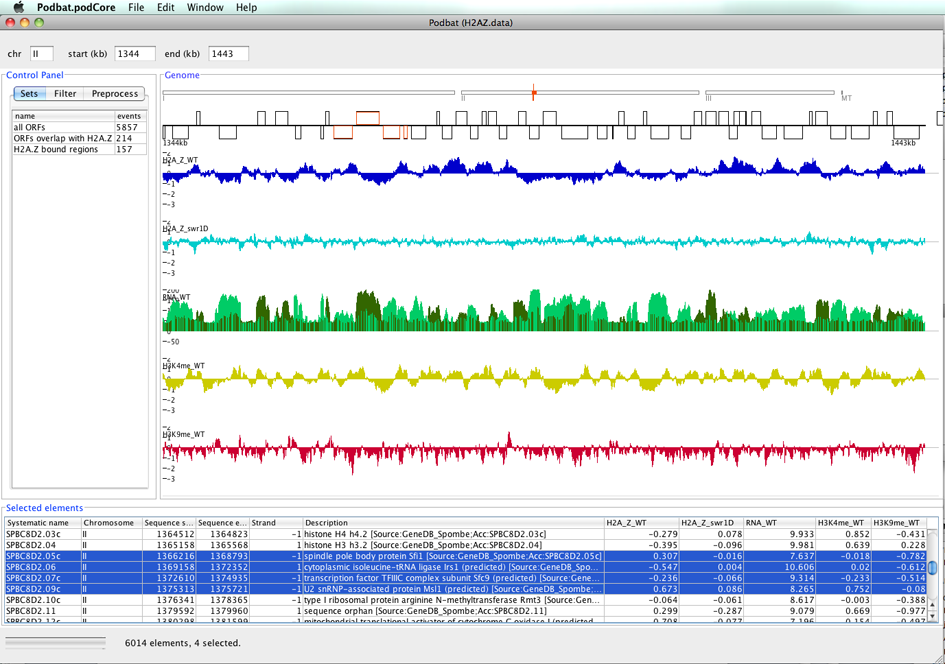

Supplement: Figure S1 — Screenshot of Podbat with 5 datasets loaded. Four genes on chromosome II are highlighted in red. A quantification of the signal has been determined of the protein binding/RNA expression/histone modification. (TIF) [file pcbi.1002163.s001.tif]

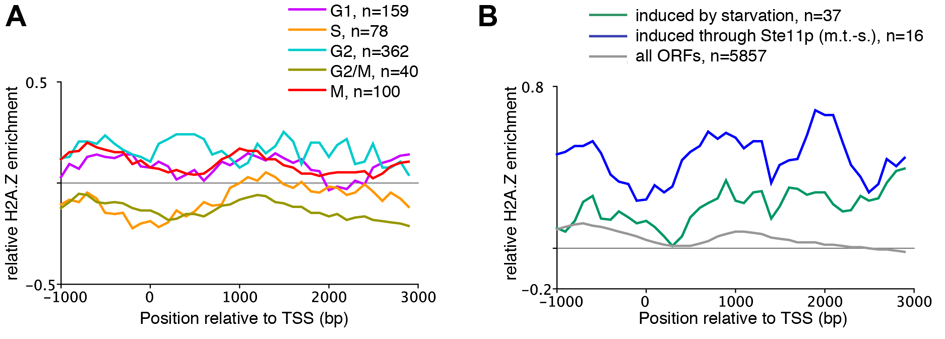

Supplement: Figure S2 — H2A.Z bound genes of different gene sets are aligned at the Translation Start Site (TSS) from the dataset GSM432595 from Zofall et al. [10]. H2A.Z bound genes were identified as overlapping with HMM determined regions of increased H2A.Z signal (parameters used as in the other datasets with the exception of Region length threshold = 20 instead of 100, since this array has probes more sparsely spaced). 94 regions were identified, overlapping with 438 genes. A) Genes differentially expressed during different stages of the cell cycle [16]. B) Gene induced by late after nitrogen starvation [12] and mating-type specifically (m.t.-s.) regulated genes induced through Ste11p [14]. (TIF) [file pcbi.1002163.s002.tif]

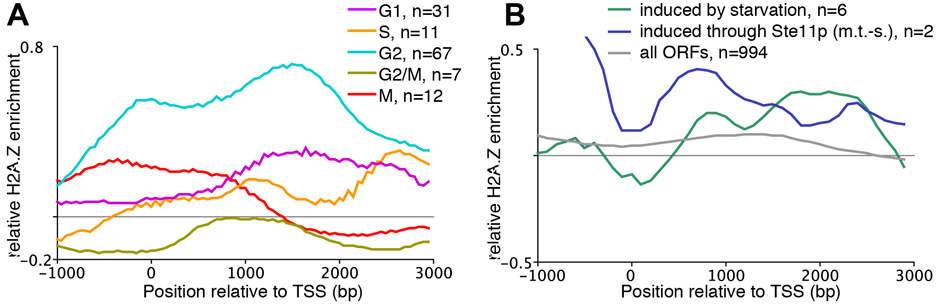

Supplement: Figure S3 — H2A.Z bound genes of different gene sets are aligned at the Translation Start Site (TSS) from the dataset GSM432576 of the partial from Zofall et al. [10]. Only approximately 2 Mb of the genome (15%), consiting of chromosome II and three telomeric sequences (1L, 2L, 2R) are covered in this array. 994/5857 ORFs are within this region. H2A.Z bound genes were identified as overlapping with HMM determined regions of increased H2A.Z signal (parameters used as in the other datasets). 27 regions were identified, overlapping with 113 genes. A) Genes differentially expressed during different stages of the cell cycle [16]. B) Gene induced by late after nitrogen starvation [12] and mating-type specifically (m.t.-s.) regulated genes induced through Ste11p [14]. NB the small number of investigated genes in these gene set. (TIF) [file pcbi.1002163.s003.tif]

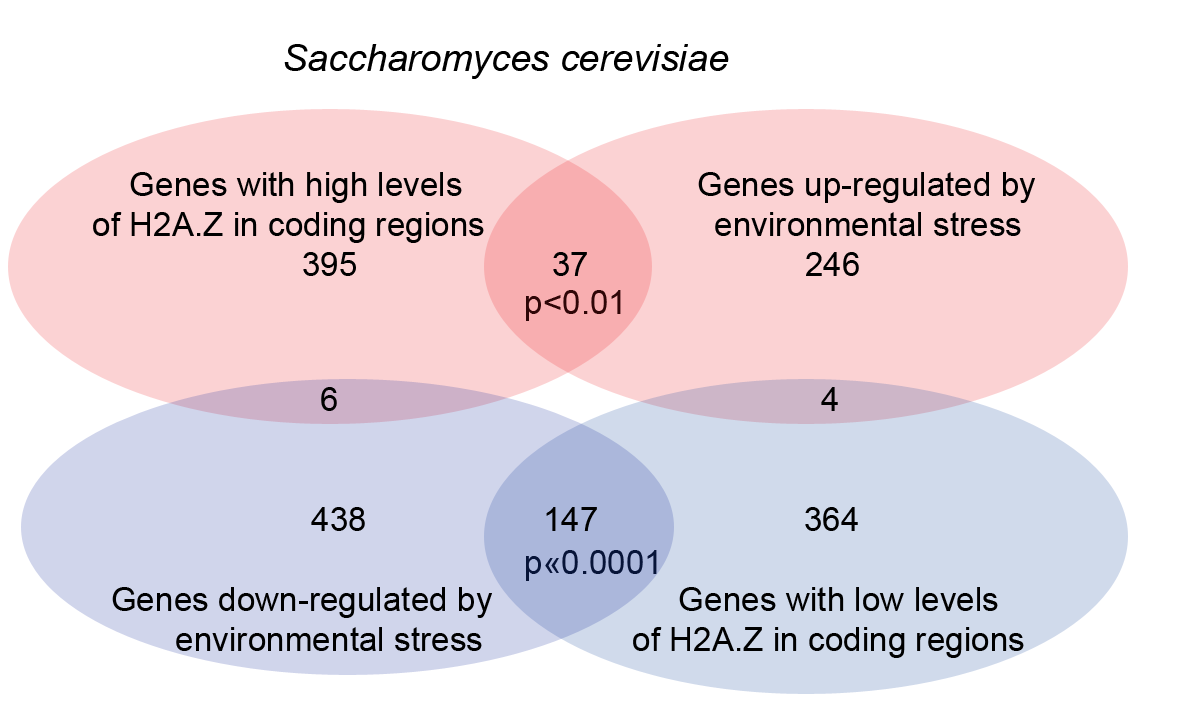

Supplement: Figure S4 — Venn diagrams showing the overlap between genes where the coding regions are bound by H2A.Z homolog in S. cerevisiae (levels deviating 2-fold from genome average) and genes differentially regulated after environmental stress. P-values for overlaps are calculated by the hypergeometric distribution. (TIF) [file pcbi.1002163.s004.tif]
