# Supplementary material for: Podbat: A Novel Genomic Tool Reveals Swr1-Independent H2A.Z Incorporation at Gene Coding Sequences through Epigenetic Meta-Analysis
Source: PLoS Comput Biol. 2011 Aug 25;7(8):e1002163. doi: 10.1371/journal.pcbi.1002163 (PMC3161910; doi:10.1371/journal.pcbi.1002163)
Supplement: Software S1 — Podbat software. (TAR) [file pcbi.1002163.s005.tar › podbat05/Podbat Instructions.pdf]

Instructions to regenerate analysis

Podbat: A Novel Genomic Tool Reveals Swr1-independent H2A.Z Incorporation at Gene Coding Sequences Through Epigenetic Meta Analysis

Laia Sadeghi, Carolina Bonilla, Annelie Strålfors, Karl Ekwall, J. Peter Svensson

First, install Podbat and load data.

i. Untar the file "Podbat.tar".
ii. Double-click on the podbat java icon to open Podbat.
iii. Import new data from the central database (**File** > **Import** > **Data from database**, figure 1)

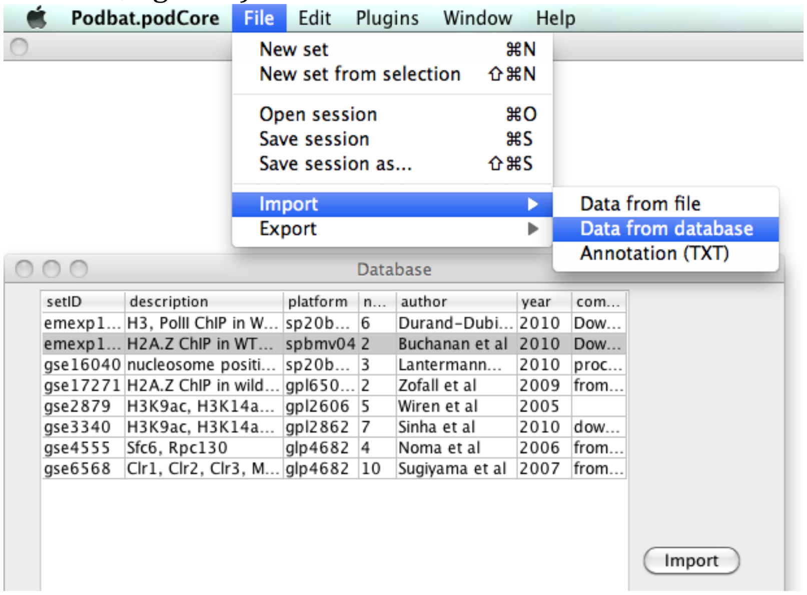

Figure 1. Load H2A.Z data from the central database.

If opening fails because of memory issues, try opening Podbat from command line. If you cannot connect to the central database, go to http://www.podbat.org and download session files "H2AZ.data" or the smaller "H2AZ_Small.data. Open the session "H2AZ.data" (**File** > **Open Session**, locate the folder containing the downloaded files, Figure 2).

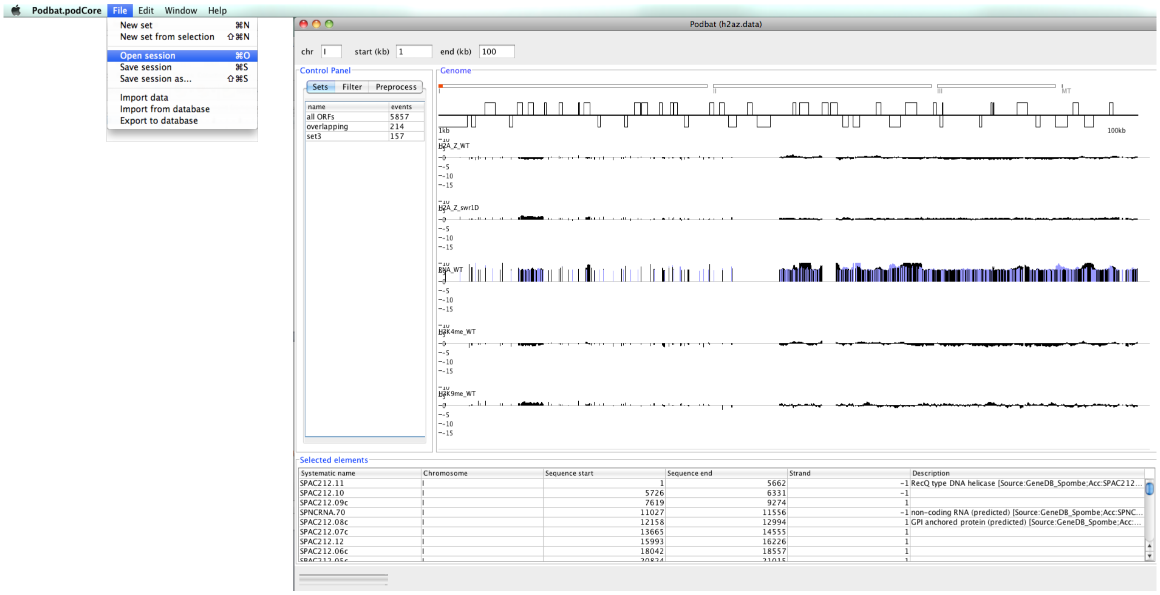

Figure 2. Open the session with the H2A.Z data.

To regenerate Figure 1A:

Colors and scales in the sample display can be adjusted by clicking on the sample data.

To estimate regions of H2A.Z binding, go to the Preprocess tab in the Control panel. Click the button to Determine regions (Figure 2).

The panel will expand. Select 'H2A.Z_WT' from the dropdown menu. Click Advanced (Adv) to access the HMM parameters and to determine them by the Baum-Welch algorithm. Click … and then OK. Now find the regions by clicking 'Find'.

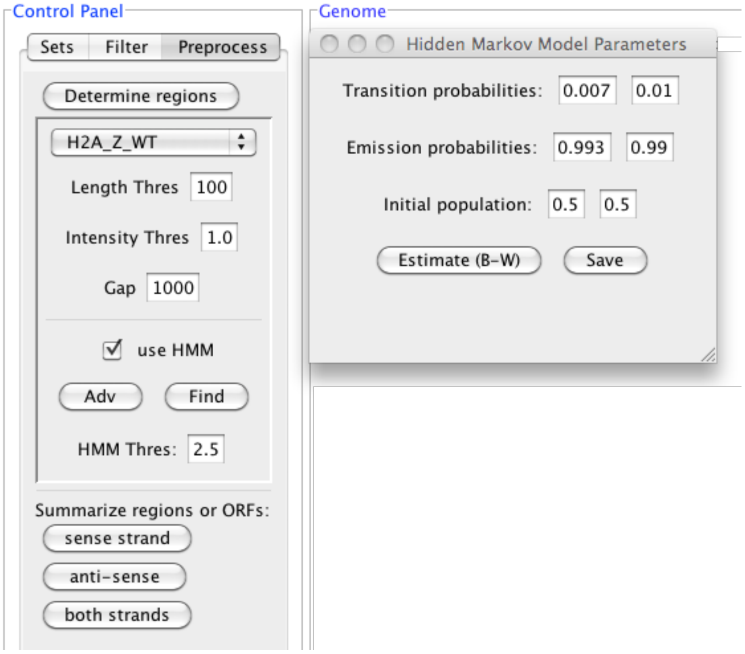

Figure 2. Identifying regions with higher H2A.Z occupancy by Hidden Markov Models (HMM).

Two new gene sets will appear in the Sets tab of the Control panel, one with the regions and one with the overlapping ORFs.

To visualize the H2A.Z binding pattern across the regions or ORFs aligned after the Translation Start Site, go to the menu and **Edit** > **Average gene plot** (Figure 3). For a default view, click Draw.

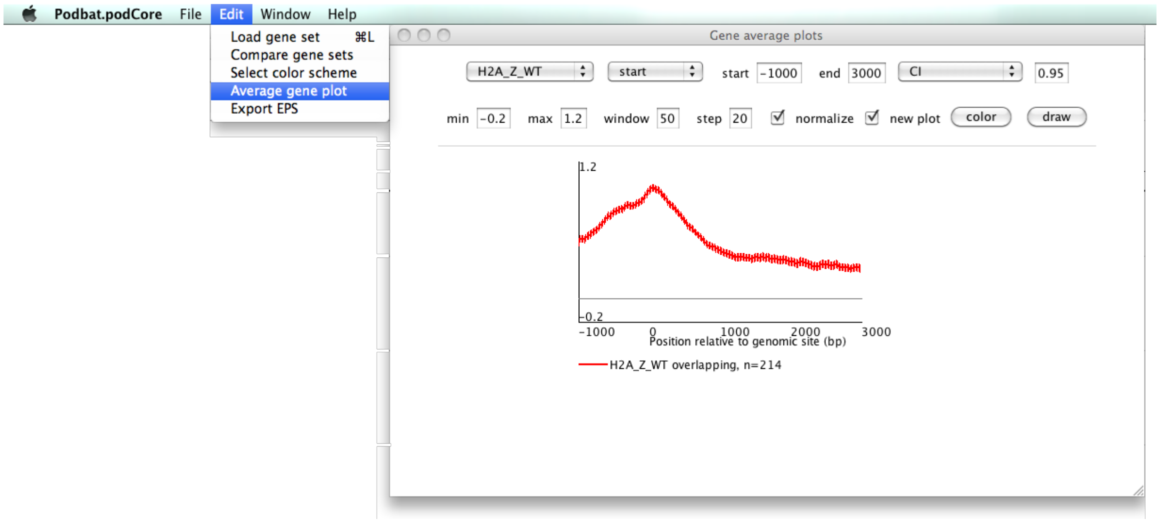

Figure 3. Aligning the genes in a gene set after their start site.

To regenerate Table 2:

Go to the menu, **Edit** > **Compare lists**. Again, in the new menu, go to **Gene sets** > **Activate database (GO, other)** (Figure 4). Locate and open the file with the Gene Ontology gene set description (schizosaccharomyces_pombe_GO.txt) in the 'data' subfolder within the Podbat folder.

Select the set "overlapping genes" and click 'Calc enrichment'. In a new tab in the spreadsheet, a table will appear, which can be sorted e.g. on the p-values. To further analyze and filter out small gene sets, the spreadsheet data can be copied and pasted into e.g. Excel.

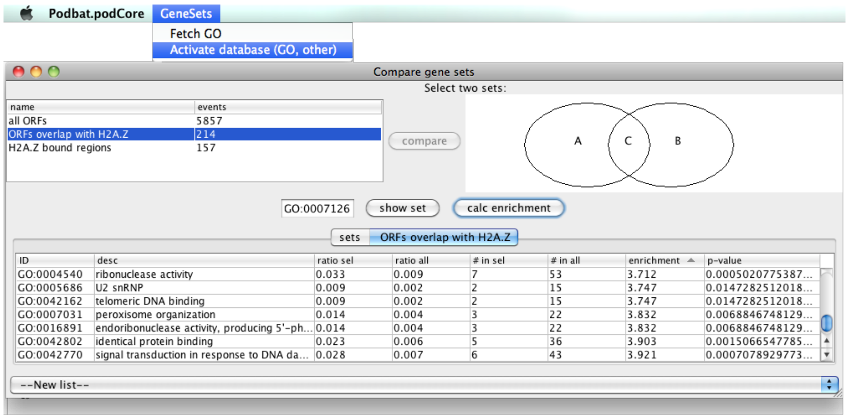

Figure 4. Activating the GO database to quantify functional enrichment within gene sets.

To regenerate Table 3 and Figure 3A,B and 4A:

Go to the menu, **Edit** > **Compare lists**. Again, in the new menu, go to **Gene sets** > **Activate database (GO, other)**. Locate and open the file with the literature gene set description (genesets_from_literature.txt) in the 'data' subfolder within the Podbat folder.

Select a gene set (e.g. "overlapping genes") and click the button 'calc enrichment'. Go to the newly created spreadsheet tab. Click on any of the gene sets and see that the ID appears in the text field next to the button 'show set' (Figure 5). Click the 'show set' button and the set will appear in the list of gene sets (both in the 'Compare gene set' window and in the Control panel of the main window.

To visualize the H2A.Z binding pattern surrounding the gene start (or end) of the gene set, select the gene set in the control panel and then open the Gene plot window (go to the menu and **Edit** > **Average gene plot**). Click the 'draw' button. To draw another gene set in the same graph, deselect the 'new plot' box and select another gene set in the control panel of the main window. Click the draw button again and the new pattern will appear.

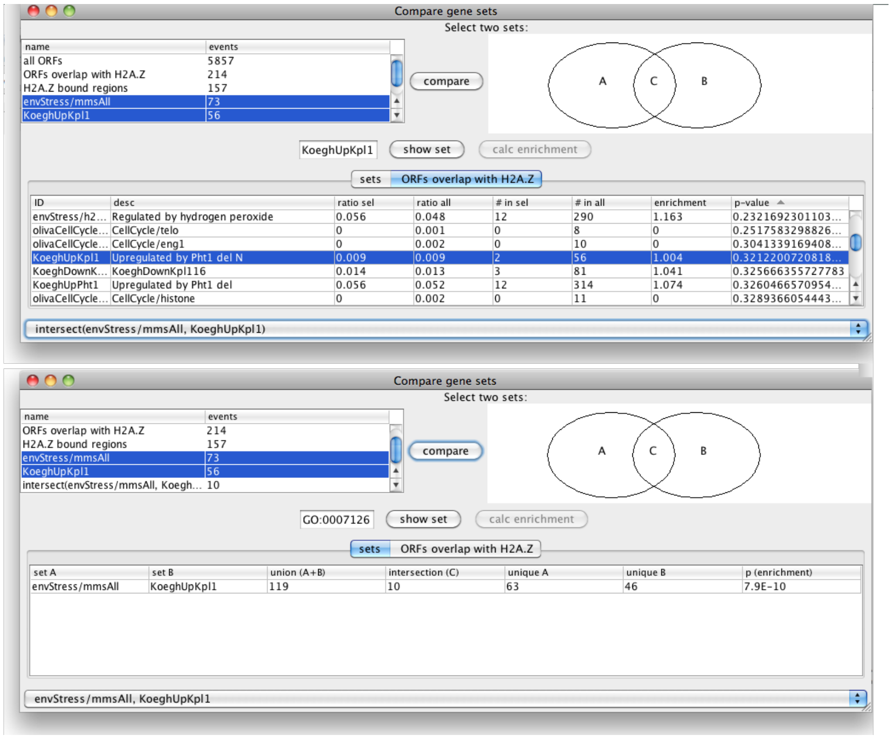

Figure 5. Comparing gene sets. Top) Extracting new gene sets from the spreadsheet. Bottom) Comparing two of the gene sets with regards to common and unique genes.

To regenerate data for Figure 3C and 4B:

To compare gene lists and get data for Venn diagrams, open the ‘Compare gene sets’ window. Select two gene sets to compare and click the ‘compare’ button. In the ‘sets’ tab of the spreadsheet, the number of genes in common in both gene sets and the unique genes will appear. Through the drop-down menu, these new gene sets can be imported into the main window.

To regenerate supplemental data

To load the datasets by Zofall et al, go to the menu, **File** > **Import data**. Select the file ‘GSM432595_signal.txt’ from the data folder (Figure 6). Repeat and select the other file ‘GSM432576_signal.txt’.

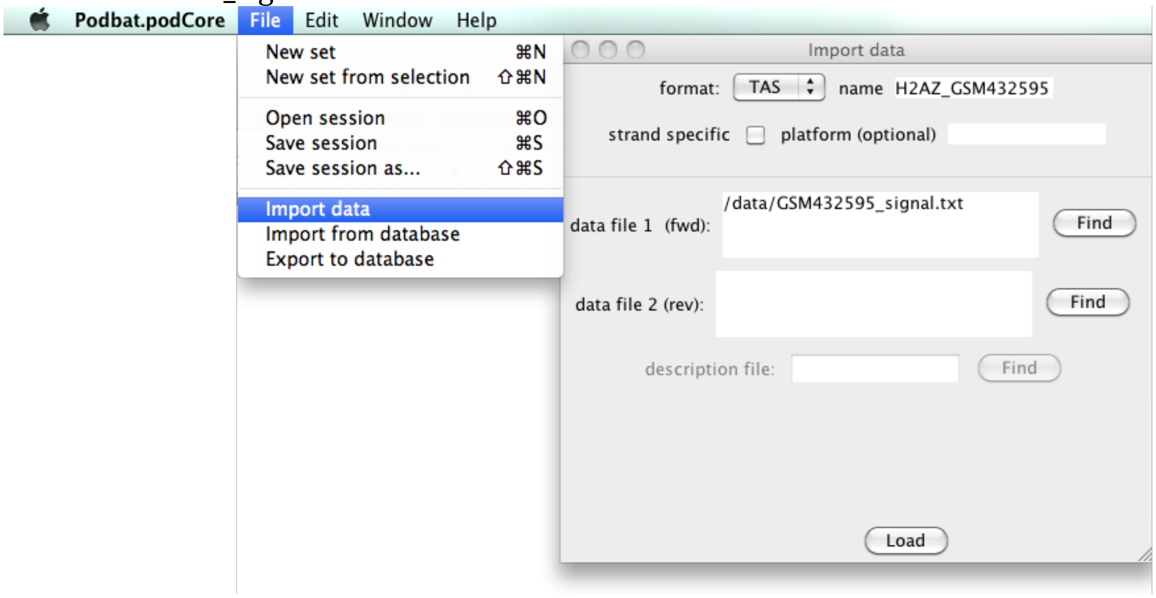

Figure 6. Importing data from text files.

Further assistance

For help on further analysis, please go to the online manual (www.podbat.org).
